# Targets preliminary screening for the fresh natural drug molecule based on Cosine-correlation and similarity-comparison of local network

**DOI:** 10.1186/s12967-022-03279-w

**Published:** 2022-02-03

**Authors:** Pengcheng Zhao, Lin Lin, Mozheng Wu, Lili Wang, Qi Geng, Li Li, Ning Zhao, Jianyu Shi, Cheng Lu

**Affiliations:** 1 School of Life Science, Northwestern Polytechnical University, Xi’an, 710072 China; 2 Institute of Basic Research in Clinical MedicineChina Academy of Chinese Medical Sciences, Beijing, 100700 China; 3 Institute of Acupuncture and Moxibustion, China Academy of Chinese Medical, Beijing, 100700 China; 4 Chemical and Biological Engineering, Yichun University, Yichun, 336000 China

**Keywords:** Targets screening, Fresh natural drug molecule, Cosine-correlation, Similarity-comparison, Western-Blot

## Abstract

**Background:**

Chinese herbal medicine is made up of hundreds of natural drug molecules and has played a major role in traditional Chinese medicine (TCM) for several thousand years. Therefore, it is of great significance to study the target of natural drug molecules for exploring the mechanism of treating diseases with TCM. However, it is very difficult to determine the targets of a fresh natural drug molecule due to the complexity of the interaction between drug molecules and targets. Compared with traditional biological experiments, the computational method has the advantages of less time and low cost for targets screening, but it remains many great challenges, especially for the molecules without social ties.

**Methods:**

This study proposed a novel method based on the Cosine-correlation and Similarity-comparison of Local Network (CSLN) to perform the preliminary screening of targets for the fresh natural drug molecules and assign weights to them through a trained parameter.

**Results:**

The performance of CSLN is superior to the popular drug-target-interaction (DTI) prediction model GRGMF on the gold standard data in the condition that is drug molecules are the objects for training and testing. Moreover, CSLN showed excellent ability in checking the targets screening performance for a fresh-natural-drug-molecule (scenario simulation) on the TCMSP (13 positive samples in top20), meanwhile, Western-Blot also further verified the accuracy of CSLN.

**Conclusions:**

In summary, the results suggest that CSLN can be used as an alternative strategy for screening targets of fresh natural drug molecules.

## Background

Traditional Chinese medicine (TCM) is an important part of the medical system in China, and its systematic and holistic view of treating diseases has been increasingly valued by the scientific community [1]. Therefore, it is of great significance to explain the overall mechanism of TCM's function for the sake of promoting modernization of TCM and development of modern medicine. The microscopic manifestation of the overall efficacy of TCM is that it forms a complex network of multiple natural drug molecules to act on multiple targets and produce synergistic effects in different pathways and functional modules, so the most pressing task is to uncover the truth of this phenomenon[2]. However, the discovering process is still both time-consuming and costly in the biological experiments [3, 4].

In the early stage, when a fresh-natural-drug-molecule was found from nature, some researchers identified drug-target interaction from literature through text mining technique [5, 6] and explored drug-target interaction through common biological elements of drug and target [7, 8]. They applied some methods based on text mining to collect the known drug-target interaction from literature, but they could not predict the new interactions. In fact, a large number of drug molecules and targets have no common elements [9], which also reduces the ability of text mining methods to recognize DTIs.

Over the past few decades, many researchers have made predictions of the interaction between targets and drug molecules based on the available data [10, 11], which contributed a lot to drug discovery and drug recycle in other situations. For example, Chen et al. [12] creatively used unsupervised pre-training and supervised fine-tuning to predict associations of miRNA-disease. Lee et al. [13] constructed a directed network of protein interactions and gene data, consequently inferred the shortest path between targets and genes. Lu et al. [14] investigated the predictive power of similarity indices such as common neighbors and Jaccard Index on predicting DTI, purely based on known DTI information.

Although machine learning methods had been proposed for drug-target interactions prediction, the predictive performance of many methods needs to be improved. First, a large number of methods were adopted on basis of the characteristics of drugs and targets with the known drug-target correlations to predict DTIs. However, not all drugs and targets have complete characteristics. If the information is incomplete, the prediction method cannot be effectively predicted. Second, some researchers found that the traditional similarity-based methods are effective for specific protein classes, but not for other classes [11]. On the other hand, almost all algorithms, whose purpose is to find targets for the drug molecules that had been studied, are designed based on the drug molecules' social relationships, they can't provide services for a fresh natural drug molecule that has no interactions with any target. But drug development has more needs for that aspect, in other words we need a predictor to screen the targets for a fresh-natural-drug-molecule when it is separated from the medicinal plants or animals.

Cosine-correlation is an algorithm that measures the difference between two individuals by the cosine of the Angle between two vectors in the vector space. It possesses the characteristics of high reliability and simple operation and has been used in many kinds of scientific researches, in particular, it tends to perform better when the input vector is sparsely populated and high-dimensional [15–18]. Notably, the fingerprints of drug molecules are generally high dimensional and sparse vectors.

Hence, on the grounds of the idea that is molecules, which bound to the same target, have similar structures [19], a computation method screening the possible targets for the fresh natural drug molecules based on the Cosine-correlation and Similarity-comparison of Local Network (CSLN) was proposed in this paper, it can perform its target screening for a molecule newly discovered in nature, even if it has no known interaction with any targets.

The traditional Chinese medicine systems pharmacology database and analysis platform (TCMSP) was built based on the framework of systems pharmacology for herbal medicines [20]. It is a relatively comprehensive database for collecting relevant data in the field of TCM, it is designed to fuel the development of herbal medicines and to promote the integration of modern medicine and traditional medicine in drug discovery and development.

In addition, since triptolide is a very widely studied natural drug molecule [21], we used CSLN to screen targets for triptolide (simulating the situation of a fresh natural drug molecule), the train and test set were constructed through TCMSP. Meanwhile, Western-Blot (WB) [22] was used to verify the screening results.

## Materials and methods

### Materials

The CSLN mainly uses the following databases for experiments and verification. We acquired four datasets of the DTI network from the gold standard data [23] coovering nuclear receptors (NR), enzymes (EN), G-protein coupled receptors (GPCR) and ion channels (IC). And it can be downloaded from http://web.kuicr.kyoto-u.ac.jp/supp/yoshi/drugtarget/. Each dataset includes 2 types of information, the observed DTIs and the similarities among drugs. In addition, the detailed statistics of the above four datasets are shown in Table 1.

**Table 1:** The statistics of the four datasets of gold standard data

	NR	EN	GPCR	IC
Numbers of Targets	26	664	95	204
Numbers of Drugs	54	445	223	210
Numbers of Interactions	90	2926	635	1476
Sparsity(%)	93.59	99.01	97.00	96.55

### Cosine-correlation and similarity-comparison of Local Network (CSLN)

**Fig. 1:**
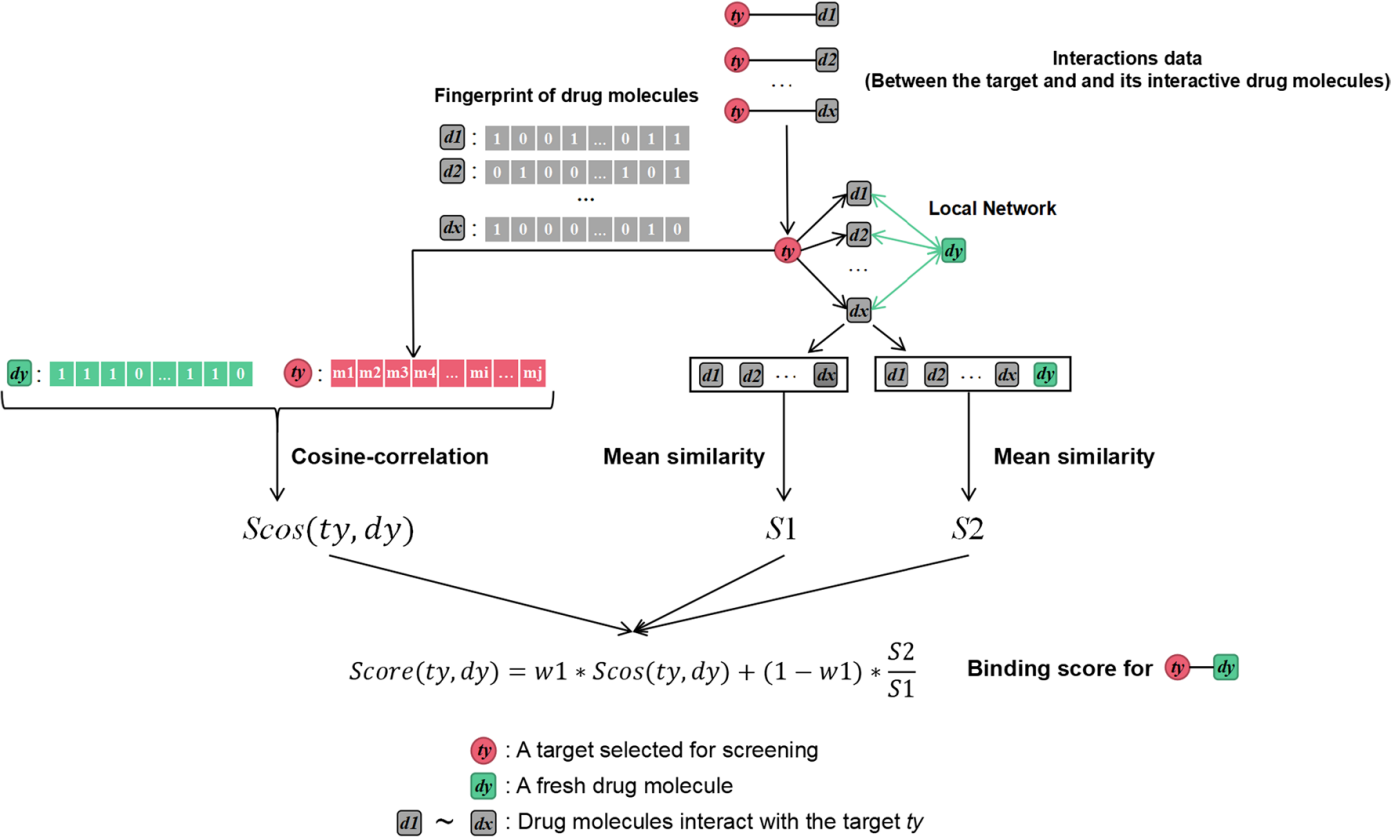
The overall architecture of CSLN [1]. Get the molecular fingerprint through MACCSkeys based on Rdkit; [2] Tanimoto was used to calculate the similarity between two molecules; [3] *w*1 is a globally shared value trained from the training dataset

The specific implementation process of CSLN is shown in Fig. 1, which can be subdivided into the following steps. First, we got the molecular fingerprint through MACCSkeys based on Rdkit [24] for all drug molecules. Second, Tanimoto [25] was used to calculate the similarity between two molecules. Third, the screened target was expressed in combination with related drugs, and its similarity with the fresh drug molecule was calculated with Cosine-correlation [16]. Fourth, compared the average similarity of local networks after the addition of the fresh drug molecule with that before the addition. Finally, the binding score of the target to the fresh drug molecule was obtained by combining the two values and assigning different weights to them through negative feedback adjustment in machine learning. Then the scores were ranked from high to low, and the predicting results of the higher score were more likely to be the potential DTIs.

The drug-target dataset was described as a binary network $V=\left(D,T,A\right)$. $D=\left\{d1, d2, ..., dm\right\}$ was a collection of drug nodes, $T=\left\{t1, t2, ..., tn\right\}$ was the set of target nodes and $A=\left\{a11, ..., aij, ..., amn\right\}$ was the set of edges between interconnected nodes in the network, where D and T respectively represented two independent sets. If there was a known interaction between the drug di and the target $tj$, then set $aij = 1$, otherwise set $aij = 0$.

Based on RDKit, the characteristics of chemical molecules were expressed in binary. The MaccsKeys fingerprint was put forward by a company whose name is MDL and had a total of 166 features, but the total length of MaccsKeys was 167bits, because bit 0 was a placeholder, and bit 1–166 was a molecular feature bit. Then, the drug molecule *di* can be expressed as:

$$Fdi=\left\{fdi0,fdi1,fdi2,\dots ,fdi166\right\}$$

The Tanimoto score between the drug molecule $di$ and $dj$ is calculated according to the following formula:

$$TN(di,dj)=\frac{Fdi\cap Fdj}{Fdi\cup Fdj}$$

where:

$Fdi$ is the elements of molecular fingerprint of $di$;

$Fdj$ is the elements of molecular fingerprint of $dj$;

For example, we calculate the binding score between a target $ty$ and a fresh drug molecule $dy$, the drug molecules $d1, d2, ..., dx$ are which interact with$ty$.

Here, $d1, d2, ..., dx$ together denote$ty$:

$$Fty=\left\{fty0,fty1,fty2,\dots ,fty166\right\}=\left[\sum_{m=1}^{x}fdm0,\sum_{m=1}^{x}fdm1,\sum_{m=1}^{x}fdm2,\dots ,\sum_{m=1}^{x}fdm166\right]$$

Cosine-correlation of $ty$ and $dy$

$$Scos\left(ty,dy\right)=\frac{{\varvec{F}}{\varvec{t}}{\varvec{y}}\bullet {\varvec{F}}{\varvec{d}}{\varvec{y}}}{\Vert {\varvec{F}}{\varvec{t}}{\varvec{y}}\Vert \Vert {\varvec{F}}{\varvec{d}}{\varvec{y}}\Vert }=\frac{\sum_{i=0}^{166}ftyi*fdyi}{\sqrt{\sum_{i=0}^{166}ftyi}*\sqrt{\sum_{i=0}^{166}fdyi}}$$

Then, the mean similarity of drug molecules ($d1, d2, \dots \dots , dx$) *S1* is calculated by the following formula

$$S1=\frac{\sum_{i=1}^{x-1}\sum_{j=i+1}^{x}TN(di,dj)}{\frac{\mathrm{x}(\mathrm{x}-1)}{2}}$$

And, the mean similarity *S2* is calculated according to the following formula when $dy$ is merged with the drug molecules

$$S2=\frac{\sum_{i=1}^{x-1}\sum_{j=i+1}^{x}TN(di,dj)+\sum_{i=1}^{x}TN(di,dy)}{\frac{\mathrm{x}(\mathrm{x}+1)}{2}}$$

where

$TN(a, b)$ represents the similarity between drug molecule *a* and drug molecule *b* (Obtained through Tanimoto);

Finally, we can get the binding score of target $ty$ with drug $dy$ according to the formula

$$Score\left(ty,dy\right)=w1*Scos\left(ty,dy\right)+\left(1-w1\right)*\frac{S2}{S1}$$

where *w*1 (Global Shared) is the weight value between the Cosine-correlation and the Similarity-comparison scores. And they are obtained through feedback learning in training. The calculation formula of residual error in feedback learning is as follows

$$Error=\sum_{i=1}^{n}\left|{y}_{i}-{\widehat{y}}_{i}\right|$$

where ${y}_{i}$ is the true label of the sample, and ${\widehat{y}}_{i}$ is the predicted value.

### Case study and verification

In the past decades, triptolide, a very widely studied natural drug molecule, has attracted considerable interest in the organic and medicinal chemistry society owing to its intriguing structural features and promising multiple pharmacological activities. However, its imprecise mechanism of action and severe toxicity have greatly hindered its clinical potential [21]. Therefore, in this study, triptolide was selected as the experimental object to predict its targets by CSLN and TCMSP was used to build the training set and test set. Notably, the environment of new natural drug molecules was simulated in this experiment.

### Cell culture

The HL-7702 cell lines and L02 cells were obtained from China Academy of Chinese Medical Sciences (Beijing, China). L02 cells were cultured in Dulbecco’s Modified Eagle’s Medium (DMEM, Sigma, USA) supplemented with 10% fetal bovine serum (Solarbio, Beijing, China), 100 units/ml penicillin and 100 mg/ml streptomycin (Solarbio, Beijing, China). All cell lines were in a humidified environment containing 5% CO_2_ at 37 °C.

### Western-Blot

The indicated cells were lysed in RIPA buffer (solarbio, Beijing, China) at 4 °C. The protein concentration was determined according to BCA Protein Assay Kit (solarbio, Beijing, China), and the equal amounts of cell lysates (30–50 µg of proteins) were subjected to 10% SDS-PAGE. After electrophoresis, proteins were transferred onto PVDF membrane Then the membranes were blocked with 5% BSA at room temperature for 1 h, followed by the incubation with NRH dehydrogenase [quinone] 2(NQO2) (1: 1000; ProteintechGroup, Inc, Beijing, China) and β-actin (1:1000; ProteintechGroup, Inc, Beijing, China) antibody at 4 °C overnight. After the incubation with the primary antibodies, the membranes were rinsed and probed with HRP-conjugated Affinipure Goat Anti-Rabbit IgG(H + L) (1:5000; ProteintechGroup, Inc, Beijing, China) for 1.5 h at room temperature. Then the immune-reactive bands were detected through ChemiDocTM Touch Imaging System (Bio-RAD, USA), and ImageJ software was used to analyze the gray value of the strip.

### Statistical analysis

All data in this experiment were expressed as the mean ± SEM values. Multiple statistical analyses were conducted with one-way analysis of variance (ANOVA). A probability value of *p* < 0.05 was defined as significant. GraphPad Prism6.0 was used for statistical analyses.

## Results

To systematically evaluate the performance of the method in every dataset of gold standard data, tenfold cross-validation was used to evaluate the generalization ability of CSLN. The experimental dataset was divided into 10 parts, one ample set was randomly selected for testing, and the remaining nine sample sets were used for training. Remarkably, CSLN was an inductive method, which means that when we split data, the object was drug molecules.

When CSLN was in operation, it was necessary to set a threshold value to filter out some targets with low connection degree when comparing-similarity of local networks. In other words, targets with lower connection degrees should be deleted when constructing the training set and test set. This threshold was determined by the training set, therefore, in tenfold-cross-validation, each training set had an optimal threshold. And the way to gain the threshold in each training set was to obtain the changing relationship between the performance of CSLN and the threshold in this data set by Leave-One-Out, finally, we selected the optimal threshold according to its performance. Where Leave-One-Out referred to taking every drug molecule in the training set as the test drug, deleting all its edges in the training set, and taking the remaining data as the basic data to calculate the binding score of the drug molecule with other targets according to CSLN.

**Fig. 2:**
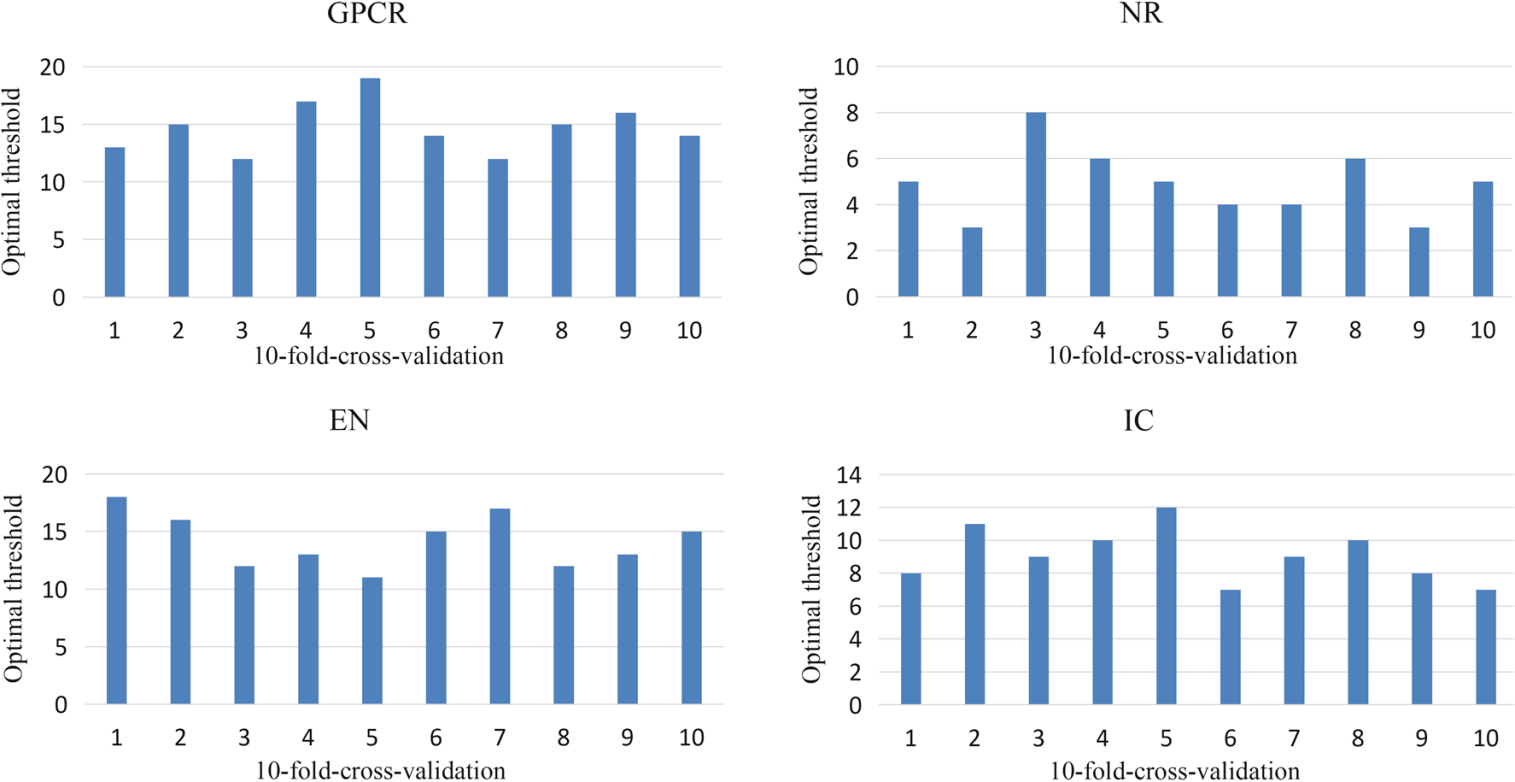
The optimal thresholds for each training set in the tenfold-cross-validation

Here, Fig. 2 shows the optimal thresholds for each training set in the tenfold cross-validation.

### Performance comparison

**Fig. 3:**
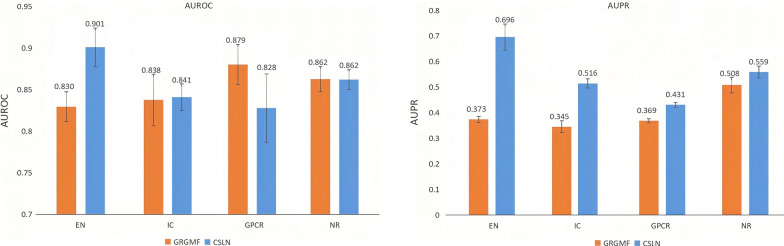
The performance comparison of DTI prediction across four datasets between CSLN and GRGMF

We mainly compared CSLN with GRGMF [26], the state-of-the-art approach which was published in 2020 and GRGMF has demonstrated superior performance over previously published models in biomedical networks. GRGMF formulated a GMF model which learns the latent factor of each node based on its neighborhood information. And instead of utilizing the similarity matrices derived from external-related databases with predefined metrics, this model could learn the neighborhood information for each node adaptively and further promote the prediction of potential links. And there is no threshold screening for GRGMF because according to the description of the author of GRGMF in the article, it is the result of calculation after decomposition of the whole matrix. If threshold screening is carried out, the information in the whole network will be reduced, so the accuracy will be reduced Here, we mainly compared the AUROC(area under ROC curve) and AUPR(area under the precision-recall) performance of CSLN and GRGMF on gold standard data, and showing the results in Fig. 3. In the results of performance comparison, CSLN's AUROC on EN and IC datasets is superior to GRGMF, meanwhile, for AUPR, the former performs better than the latter in all four data sets.

### Prediction by CSLN

**Fig. 4:**
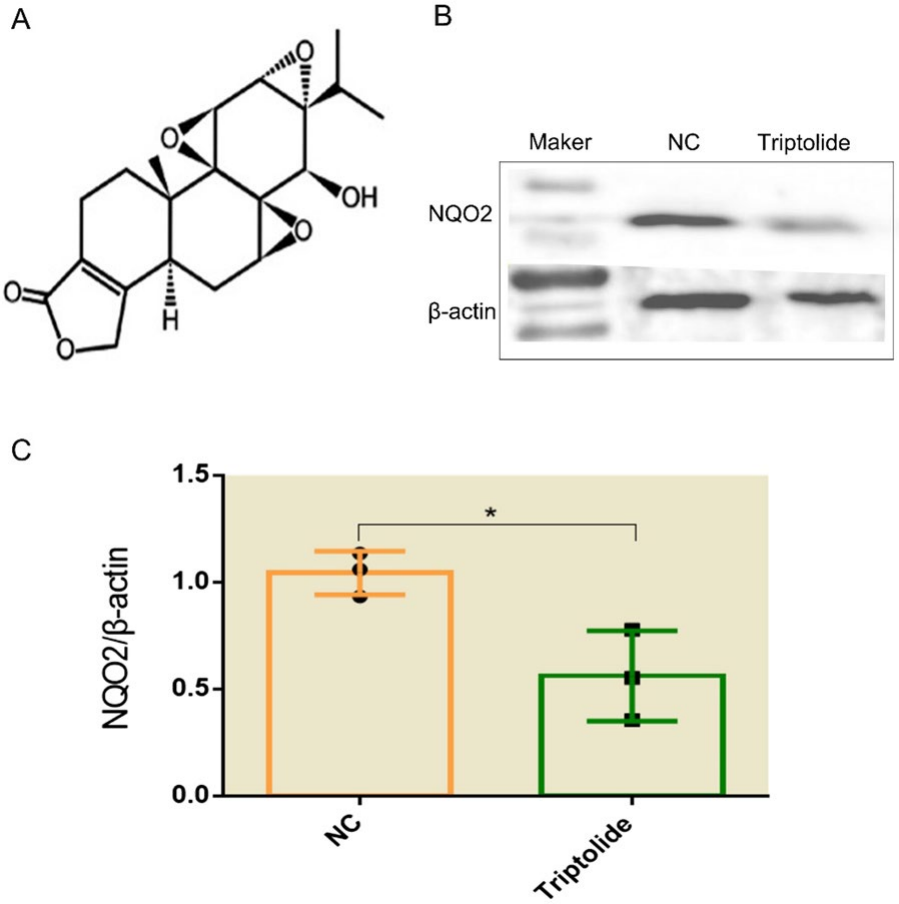
Experimental result of Western blot (*p < 0.05). **a** is the chemical formula for triptolide. **b** is the result of Western-Blot to verify the effect of triptolide on the expression of NQO2 in the L02 hepatocyte. **c** is the result of one-way ANOVA between the control group (NC) and the experimental group (Triptolide)

Further, to demonstrate the reliability of CSLN in targets prediction for fresh natural drug molecules, we did an experiment and took the triptolide (Fig. 4a) as its object. We simulated the environment of a fresh natural drug molecule by deleting its interaction with targets in the database. Meanwhile, from the results, we selected a protein with a high score that had not been found to interact with triptolide in previous relevant work to see whether triptolide could affect its expression with Western-Blot (WB).

We collected data of natural drug molecules from TCMSP (https://tcmsp-e.com/), which includes 6,494 natural drug molecules and 1,718 targets that have interactions with them, and the number of interactions is 54,852. Since triptolide is not a fresh natural drug molecule, the interactions data of it in TCMSP were deleted to simulate the situation of a fresh natural drug molecule and construct a new data-set, and CSLN was used to calculate the binding score between triptolide and the targets in the new dataset. When reconstructing the data-set, we deleted the triptolide known interactions [34] in the data-set, among which 3 targets only interacted with triptolide, to simulated the environment of a fresh natural drug molecule for triptolide. Therefore, 34 interactions and 3 targets were deleted. The detailed statistics of the above two datasets are shown in Table 2.

**Table 2:** Details of the TCMSP data

	Original	Reconstructed
Numbers of ingredients	6494	6493
Numbers of targets	1718	1715
Numbers of interactions	54,852	54,818

In this simulated prediction, Leave-One-Out was used to detect the performance of CSLN on the reconstructed data-set, and the data-set was adjusted according to the change of the threshold value to obtain the changing relationship between the threshold value and the performance of CSLN. Finally, the optimal threshold value was selected as 24. The calculation would be skipped when the link degree of the target was less than 24. There were 152 targets (include 10 positive samples) that have been selected. CSLN was used to calculate the binding scores of triptolide with 152 targets and we ranked them according to the score, the top 20 were shown in Table 3.

**Table 3:** Top 20 targets of the binding score with triptolide

	Name of the target	Source
1	Proto-oncogene c-Fos	TCMSP
2	Interleukin-6	[27]
3	Tumor necrosis factor	TCMSP
4	Apoptosis regulator BAX	[28]
5	Vascular endothelial growth factor A	TCMSP
6	Apoptosis regulator Bcl-2	TCMSP
7	Caspase-3	TCMSP
8	Gamma-aminobutyric-acid receptor subunit alpha-4	[29]
9	NRH dehydrogenase [quinone] 2	–
10	Matrix metalloproteinase-9	Stitch
11	Glutamate receptor 2	–
12	Transcription factor AP-1	TCMSP
13	Transcription factor p65	TCMSP
14	Ig gamma-1 chain C region	–
15	Glucocorticoid receptor	Stitch
16	Gamma-aminobutyric-acid receptor alpha-5 subunit	–
17	Neuronal acetylcholine receptor subunit alpha-2	[30]
18	Neuronal acetylcholine receptor subunit alpha-7	–
19	Gamma-aminobutyric-acid receptor alpha-3 subunit	–
20	Muscarinic acetylcholine receptor M2	–

Because this experiment is the simulation of targets prediction for a fresh ingredient, the source of the interaction information between the target and triptolide is briefed as the *Source*.

As we can see from the results, the 10 positive samples scored relatively high on the whole, and 7 of them appeared at the Top 20. Furthermore, there were another 6 *false-negative samples* in the Top 20 interacting with triptolide, which had been evidenced in other databases and literature.

## Results of Western-Blot

To explore whether triptolide contributes to the regulation of the expression of NRH dehydrogenase [quinone] 2 (NQO2) in L02 hepatocyte, western blot analysis was performed to detect the expression levels of NQO2. Statistical differences between the two groups were found according to one-way ANOVA (Fig. 4c), p = 0.0236 < 0.05. This indicates that triptolide reduces the expression of NRH dehydrogenase [quinone] 2 in the L02 hepatocyte (Fig. 4b).

## Discussion

In this study, we proposed a CSLN-based target screening method, which calculated the binding score between the target and the drug molecule according to cosine-correlation and similarity-comparison of the Local Network. The innovation of CSLN is that the method could predict the target for a fresh drug molecule, that is, for the newly discovered drug molecules, the possible target can be recommended more accurately with CSLN. Its advantage lies in the fact that the prediction of a drug molecule's target is not limited by its social relationships.

Meanwhile, we compared the performance of CSLN and GRGMF on the gold standard data, and the result proved that the former has a better prediction performance than the latter for the fresh drug molecules. This indicates that CSLN has a more excellent performance.

In pharmaceutical research, more fresh drug molecules are being found in nature. Therefore, we followed up with a case study of natural drug molecules. We chose triptolide, a highly studied natural drug molecule, as an object to verify the reliability of CSLN. In the target prediction experiment of triptolide, CSLN also showed excellent performance. 13 targets that have interactions with triptolide (7 of TCMSP, 6 of other databases or literature) were predicted in the top 20. These demonstrate the ability of CSLN not only to predict positive samples but also to maintain a high hit rate for potential- interactions (which didn't appear in the basal data).

In the WB experiment, we chose NRH dehydrogenase [quinone] 2 (NQO2) as the target, which had no interaction with triptolide in previous studies and it ranks the ninth in the results according to CSLN, to further verify the accuracy of CSLN. Experimental results show that compared with the blank group, the NQO2 expression quantity of the medicine group was decreased in L02 hepatocytes. This means that triptolide could down-regulate the expression of NQO2. It again proves that CSLN has high accuracy in the screening targets of fresh natural drug molecules.

Coincidentally, an interesting situation was found in the results of our validation. Qi et al. [31] found that triptolide is highly toxic and can cause toxicity to the digestive system, urinary system, blood circulation system, reproductive system, and bone marrow, causing varying degrees of damage, which seriously affects its use. In addition, according to relevant studies, renal insufficiency/failure is the most important cause of death in all cases of triptolide poisoning, and the kidney is the most important target organ for the chronic toxic effects of triptolide [32]. However, the mechanism of renal injury induced by triptolide is still unclear. Therefore, from a safety perspective, efforts must be made to understand the mechanism of the nephrotoxic effects of triptolide. Meanwhile, NQO2 is a quinone reductase associated with the conjugation of hydroquinone and is involved in detoxification pathways as well as biosynthesis processes such as vitamin K-dependent γ-carboxylation of glutamate residues in prothrombin synthesis [33]. Therefore, this suggests that, possibly, the toxic effects of triptolide are expressed through suppressing the mediation agent NQO2.

At present, the known interactions of drug-target have high sparsity, this sparsity provides unlimited possibilities for the new use of old drugs and the development of new drugs, but it leads to the low accuracy of prediction algorithms precisely, CSLN is also limited in this regard.

## Conclusion

CSLN proposed in this study performs better than GRGMF on gold standard data although GRGMF has demonstrated superior performance over previously published models in biomedical networks. In addition, when predicting the target of triptolide based on TCMSP, CSLN also performed quite accurately. Moreover, the Western-Blot experiment further proves its accuracy.

These evidence indicate that CSLN has good performance in the pre-screening stage of targets for the fresh drug molecules. Therefore, in the process of target discovery, using CSLN for pre-screening can save much time and energy for researchers. Especially, CSLN is very useful in the field of Chinese herbal medicine research. When a fresh natural drug molecule is found from plants or animals, CSLN will provide great help for ascertaining its target. It is worth noting that the final prediction result is only a binding score of the drug molecule to be predicted and the target in the data set given by CSLN, and the predicted results will be ranked according to the score. The closer the ranking is to the top, the more likely the result is to be a positive sample.

## Data Availability

The datasets generated and/or analysed during the current study are available in the gold standard data and TCMSP repository (gold standard data, http://web.kuicr.kyoto-u.ac.jp/supp/yoshi/drugtarget) (TCMSP, https://tcmsp-e.com/).

## Abbreviations

TCM: Traditional Chinese Medicine
CSLN: Cosine-correlation and Similarity-comparison of Local Network
TCMSP: The traditional Chinese Medicine Systems Pharmacology database and analysis platform
NR: Nuclear receptors
GPCR: G-Protein Coupled Receptors
IC: Ion Channels
EN: Enzymes
AUROC: Area Under the Receiver Operating Characteristic
AUPR: Area Under the Precision-Recall
WB: Western-Blot

## References

[1] Jiang M, Lu C, Chen G, Xiao C, Zha Q, Niu X (2012). Understanding the molecular mechanism of interventions in treating rheumatoid arthritis patients with corresponding traditional chinese medicine patterns based on bioinformatics approach. Evid Based Complement Alternat Med..

[2] Mao X, Xu H, Li S, Su J, Li W, Guo Q (2019). Exploring pharmacological mechanisms of Xueshuan-Xinmai-Ning tablets acting on coronary heart disease based on drug target-disease gene interaction network. Phytomedicine.

[3] Zhao N, Zheng G, Li J, Zhao HY, Lu C, Jiang M (2018). Text mining of rheumatoid arthritis and diabetes mellitus to understand the mechanisms of chinese medicine in different diseases with same treatment. Chin J Integr Med.

[4] Cao DS, Liu S, Xu QS, Lu HM, Huang JH, Hu QN (2012). Large-scale prediction of drug-target interactions using protein sequences and drug topological structures. Anal Chim Acta.

[5] Xu R, Wang Q (2013). Large-scale extraction of accurate drug-disease treatment pairs from biomedical literature for drug repurposing. BMC Bioinform.

[6] Alam F, Corazza A, Lavelli A, Zanoli R. A knowledge-poor approach to chemical-disease relation extraction. Database (Oxford). 2016;2016. 10.1093/database/baw071 PMC4869795 27189609

[7] Yang J, Li Z, Fan X, Cheng Y (2014). Drug-disease association and drug-repositioning predictions in complex diseases using causal inference-probabilistic matrix factorization. J Chem Inf Model.

[8] Zhao S, Li S (2012). A co-module approach for elucidating drug-disease associations and revealing their molecular basis. Bioinformatics.

[9] Wang L, Wang Y, Hu Q, Li S (2014). Systematic analysis of new drug indications by drug-gene-disease coherent subnetworks. CPT Pharmacometrics Syst Pharmacol..

[10] Bagherian M, Sabeti E, Wang K, Sartor MA, Nikolovska-Coleska Z, Najarian K (2021). Machine learning approaches and databases for prediction of drug-target interaction: a survey paper. Brief Bioinform.

[11] Zhang W, Yue X, Huang F, Liu R, Chen Y, Ruan C (2018). Predicting drug-disease associations and their therapeutic function based on the drug-disease association bipartite network. Methods.

[12] Chen X, Li TH, Zhao Y, Wang CC, Zhu CC (2020). Deep-belief network for predicting potential miRNA-disease associations. Brief Bioinform..

[13] Lee T, Yoon Y (2018). Drug repositioning using drug-disease vectors based on an integrated network. BMC Bioinform.

[14] Lu Y, Guo Y, Korhonen A (2017). Link prediction in drug-target interactions network using similarity indices. BMC Bioinform.

[15] Abdel-Basset M, Mohamed M, Elhoseny M, Son LH, Chiclana F, Zaied AEH (2019). Cosine similarity measures of bipolar neutrosophic set for diagnosis of bipolar disorder diseases. Artif Intell Med..

[16] Cai S, Georgakilas GK, Johnson JL, Vahedi G (2018). A cosine similarity-based method to infer variability of chromatin accessibility at the single-cell level. Front Genet.

[17] Grieb N, Oltrup T, Bende T, Leitritz MA (2020). The cosine similarity technique: a new method for smart EXCIMER laser control. Z Med Phys.

[18] Ye J (2015). Improved cosine similarity measures of simplified neutrosophic sets for medical diagnoses. Artif Intell Med.

[19] Khalili H, Godwin A, Choi JW, Lever R, Brocchini S (2012). Comparative binding of disulfide-bridged PEG-Fabs. Bioconjug Chem.

[20] Ru J, Li P, Wang J, Zhou W, Li B, Huang C (2014). TCMSP: a database of systems pharmacology for drug discovery from herbal medicines. J Cheminform.

[21] Hou W, Liu B, Xu H (2019). Triptolide: medicinal chemistry, chemical biology and clinical progress. Eur J Med Chem.

[22] Taylor SC, Posch A (2014). The design of a quantitative western blot experiment. Biomed Res Int..

[23] Yamanishi Y, Araki M, Gutteridge A, Honda W, Kanehisa M (2008). Prediction of drug-target interaction networks from the integration of chemical and genomic spaces. Bioinformatics.

[24] Bento AP, Hersey A, Felix E, Landrum G, Gaulton A, Atkinson F (2020). An open source chemical structure curation pipeline using RDKit. J Cheminform.

[25] Vogt M, Bajorath J (2017). Modeling tanimoto similarity value distributions and predicting search results. Mol Inform..

[26] Zhang ZC, Zhang XF, Wu M, Ou-Yang L, Zhao XM, Li XL (2020). A graph regularized generalized matrix factorization model for predicting links in biomedical bipartite networks. Bioinformatics.

[27] Huang Y, Chen Z, Wang Y, Ba X, Huang Y, Shen P (2019). Triptolide exerts an anti-tumor effect on nonsmall cell lung cancer cells by inhibiting activation of the IL6/STAT3 axis. Int J Mol Med.

[28] You L, Dong X, Ni B, Fu J, Yang C, Yin X (2018). Triptolide induces apoptosis through fas death and mitochondrial pathways in HepaRG Cell Line. Front Pharmacol.

[29] Xu P, Berto S, Kulkarni A, Jeong B, Joseph C, Cox KH (2021). NPAS4 regulates the transcriptional response of the suprachiasmatic nucleus to light and circadian behavior. Neuron..

[30] Wang HY, Taggi AE, Meinwald J, Wise RA, Woods AS (2005). Study of the interaction of chlorisondamine and chlorisondamine analogues with an epitope of the alpha-2 neuronal acetylcholine nicotinic receptor subunit. J Proteome Res.

[31] Qi B, Wang X, Zhou Y, Han Q, He L, Gong T (2015). A renal-targeted triptolide aminoglycoside (TPAG) conjugate for lowering systemic toxicities of triptolide. Fitoterapia.

[32] Xi C, Peng S, Wu Z, Zhou Q, Zhou J (2017). Toxicity of triptolide and the molecular mechanisms involved. Biomed Pharmacother.

[33] Long DJ, Jaiswal AK (2000). NRH:quinone oxidoreductase2 (NQO2). Chem Biol Interact.

